# Smooth Muscle-Alpha Actin Inhibits Vascular Smooth Muscle Cell Proliferation and Migration by Inhibiting Rac1 Activity

**DOI:** 10.1371/journal.pone.0155726

**Published:** 2016-05-13

**Authors:** Lihua Chen, Allison DeWispelaere, Frank Dastvan, William R. A. Osborne, Christine Blechner, Sabine Windhorst, Guenter Daum

**Affiliations:** 1 Department of Surgery and Center for Cardiovascular Biology, University of Washington, Seattle, WA, United States of America; 2 Department of Pediatrics and Diabetes and Obesity Center of Excellence at the University of Washington, Seattle, WA, United States of America; 3 Department of Biochemistry and Signal Transduction, University Medical Center Hamburg-Eppendorf, Germany; Duke University, UNITED STATES

## Abstract

Smooth muscle alpha-actin (SMA) is a marker for the contractile, non-proliferative phenotype of adult smooth muscle cells (SMCs). Upon arterial injury, expression of SMA and other structural proteins decreases and SMCs acquire a pro-migratory and proliferative phenotype. To what extent SMA regulates migration and proliferation of SMCs is unclear and putative signaling pathways involved remain to be elucidated. Here, we used lentiviral-mediated gene transfer and siRNA technology to manipulate expression of SMA in carotid mouse SMCs and studied effects of SMA. Overexpression of SMA results in decreased proliferation and migration and blunts serum-induced activation of the small GTPase Rac, but not RhoA. All inhibitory effects of SMA are rescued by expression of a constitutively active Rac1 mutant (V12rac1). Moreover, reduction of SMA expression by siRNA technology results in an increased activation of Rac. Taken together, this study identifies Rac1 as a downstream target for SMA to inhibit SMC proliferation and migration.

## Introduction

Restenosis is a major complication for all open or endovascular surgeries to restore blood flow [1,2]. A major cause for arteries to re-occlude after repair is intimal hyperplasia due to migration and excessive growth of smooth muscle cells (SMCs). In healthy arteries, SMCs highly express smooth muscle-alpha actin (SMA) and other structural and actin-binding proteins, which in their entirety define the contractile phenotype of SMCs [3–6]. In diseased or injured arteries, however, expression of these proteins in SMCs is decreased and cells acquire a less contractile but more proliferative and migratory phenotype [3–6]. Surprisingly little is known about molecular mechanisms by which structural proteins may regulate SMC proliferation and migration. Investigations of SMA-null mice, which are viable and propagate, demonstrate that SMA regulates arterial contractility and plays an important role in blood pressure homeostasis [7]. When these mice undergo vascular injury, they respond with increased neointimal hyperplasia compared to wild-type controls [8]. Isolated SMC from SMA-null arteries grow faster than controls. Suggested mechanisms include increased focal adhesion kinase activity and increased expression of platelet-derived growth factor receptor (PDGFR)-beta [8]. An inhibitory effect of SMA on cell proliferation and migration has previously also been concluded from studies with tumor cells [9,10] and fibroblasts [11], respectively. Here, we have used wild-type carotid SMCs to modulate SMA expression and found that SMA inhibits proliferation and migration by preventing the activation of Rac.

## Materials and Methods

### Ethics Statement

The preparation of smooth muscle cells from mouse arteries performed at the University of Washington was approved by the University’s Institutional Animal Care and Use Committee (IACUC) Protocol #4225–01. When performed at the University Hospital in Hamburg, the procedure has been approved by the local Department for Health and Consumer Protection, approval number ORG 680.

### Isolation and culture of mouse carotid SMCs

Carotid SMCs were isolated by protease dispersion as previously described [12] and cultured in DMEM supplemented with antibiotics (200 U/mL penicillin, 0.2 mg/mL streptomycin (all from Invitrogen-Gibco Life Technologies) and 10% fetal bovine serum (Atlantic Biologics).

### Generation of bicistronic lentivirus and cell transfection

A mouse SMA (gene name: ACTA2) cDNA clone (MGC: 76776 IMAGE: 30054569) was obtained from OriGene Technologies. The SMA cDNA was C-terminally tagged with an epitope of vesicular stomatitis virus glycoprotein G (VSVG) [13] by PCR amplification with forward 5’ primer, 5’-AGA CGA AAG CTT GAG TGG AGA AGC CCA GCC AGT-3’ and reverse 3’ primer, 5’-TCT TGG GAA TTC CTA TTA CTT ACC CAG GCG GTT CAT TTC GAT ATC AGT GTA GAA GCA TTT GCG GTG GAC-3’. The VSVG-SMA cDNA was then cloned into a bicistronic lentiviral construct also harboring GFP cDNA [14]. Lentivirus production, purification, titer determination and confirmation of replication deficiency [15] all were performed at the Virus, Molecular Biology and Cell Core which is part of Diabetes and Obesity Center of Excellence at the University of Washington. Following transfection of carotid SMCs with lentivirus overnight, cells were washed with PBS and sorted for GFP expression using a Becton Dickenson Aria II instrument. GFP- positive cells were propagated in 10% FBS until use.

### Analysis of protein expression by Western blotting

SMCs were extracted in HEPES buffer containing 1% Triton X-100 [16]. Protein was measured using Precision-Red (Cytoskeleton) and equal amounts were then subjected to SDS-polyacrylamide gel electrophoresis followed by Western blotting. Blots were developed with ECL (GE Healthcare). The following antibodies were used: anti-β-tubulin (Cell Signaling #2146), anti-smooth muscle alpha-actin (Sigma #A2547), anti-VSVG (Roche #11667351001), anti-Rac1 (BD Biosciences, #610650) and anti-RhoA (Santa Cruz sc-418). To analyze MAPK phosphorylation, serum-starved GFP-controls and SMA-SMCs were stimulated with 10% FBS and harvested at the time points indicated. Cell extracts were subjected to Western blot analysis and blots probed with antibodies to recognize the phosphorylated forms of p44/42 ERK, p38 MAPK and p54/46 JNK. All antibodies were from Cell Signaling and used at the following dilutions: #9101 (1:10,000), #9215 (1:1,000) and #9251 (1:500). To control for equal loading, blots were reprobed with HSC70 antibody (Santa Cruz, sc-7298, 1:6000).

### Immunocytochemistry

SMCs were plated at 8x10^3^ cells/well in fibronectin-coated 8-well chamber slides. Next day, cells were fixed in 4% para-formaldehyde and permeabilized with 0.1%Triton X-100 for 10 min. Following blocking with Imagine-It FX Signal Enhancer (Invitrogen), slides were incubated overnight with vinculin antibody (Sigma #V9131; 1:400) at 4°C followed by incubation with the fluorescence probe AlexaFluor 568 (Molecular probes, 1:1000). Alternatively, slides were stained for F-actin with rhodamine-coupled phalloidin (Cytoskeleton, 100 nmol/L) for 1 hour at room temperature. Fluorescence was analyzed with a Zeiss confocal microscope and images were acquired using Zeiss LSM Aim examiner software.

### Proliferation Assay

Cell replication was measured using a metabolic labeling assay as described [17]. Cells were plated into 12 well dishes at 2000 cells/well using growth media containing 10% FBS. At the appropriate time points, cells were incubated with 1 mg/mL MTT (3-(4,5-Dimethylthiazol-2-yl)-2,5-diphenyltetrazolium bromide, Sigma) at 37°C for one hour. Cells were then washed with PBS, dried, and MTT precipitate (formazan) was solubilized in 0.1 mL DMSO/well. Absorption of MTT solution was measured at 560 nm and corrected for DMSO alone. Assays were performed in triplicates.

### Wound healing (migration) assay

Cells were plated at high density into 12 well plates (8x10^5^ cells/well). Next day, the cell monolayer was wounded by scratching the plate with a pipette tip generating a cell-free line. Cell debris was washed away and cells incubated in media containing 10% FBS and hydroxyurea (5 mmol/L) to prevent cell proliferation. At this time, a photograph was taken. After 17 hours incubation at 37°C, a second photograph was taken at the same location. Cell-free areas were quantified using Adobe Photoshop CS4 software. Migration is expressed as percent coverage. Assays were performed in triplicates.

### Rac and RhoA activity assays

Rac and RhoA activities were assessed by measuring their GTP-bound form using Elisa kits from Cytoskeleton (BK125 and BK124). It should be noted that the Rac assay measures all Rac isoforms (Rac1, Rac2 and Rac3). Following stimulation of SMCs as indicated, extracts were generated and snap-frozen before subjected to the assay, which was performed as to the manufacturer’s instructions. Assays were performed in triplicates.

### Treatment of SMCs with siRNA

SMCs (7x10^5^) were resuspended in 0.1 mL aortic SMC Nucleofactor solution (Amaxa) and electroporated in the presence of 2 nmol/L control siRNA (medium GC) or siRNA targeting SMA (sense: CAGAGACUCUCUUCCAGCCAUCUUU, antisense: AAAGAUGGCUGGAAGAGAGUCUCUG) using program U-25 (Amaxa). Following transfection, cells were kept in growth media containing 10% FBS for 3 days before they were used in experiments.

### Adenoviral-mediated expression of constitutively active Rac1 (V12rac1)

Adenovirus expressing constitutively active Rac1 (V12rac1), in which glycine at position 12 in human Rac1 was replaced by valine, was a generous gift from Dr. Goldshmidt-Clermont, Ohio State University, Columbus, Ohio [18]. Controls or SMCs expressing SMA were transfected with adenovirus expressing lacZ or V12rac1 with a MOI of 100 for 30 hours. Following 1 day of recovery, cells were used for migration and growth assays.

### Statistical Analysis

Data are presented as mean +/- S.D. Statistical significance between two groups was calculated by two-tailed t-test. P>0.05 was considered non-significant.

## Results

**Fig 1:**
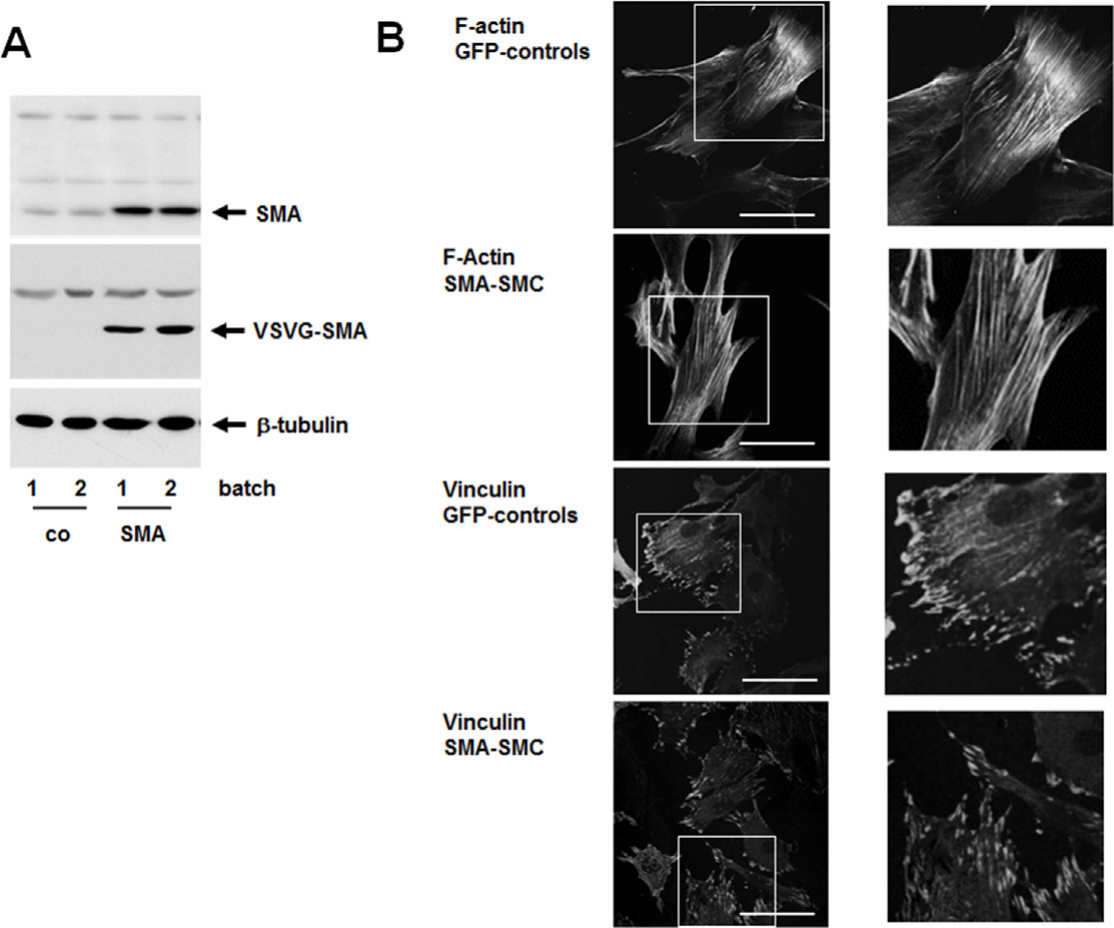
Visualization of actin fibers and focal adhesion sites in SMCs overexpressing SMA. SMCs were transfected with bicistronic lenti virus expressing GFP alone (co, GFP-control), or in addition VSVG-tagged SMA (SMA). (A) Overexpression of SMA was confirmed by Western blot analysis of cell extracts using antibodies against SMA or VSVG. Staining for β-tubulin was included as loading control. Two independent batches of cells were prepared for controls and SMA overexpression. Note the slight up-shift of the SMA band in SMA-SMC-extracts due to the VSVG-tag. (B) GFP-controls or SMA-SMCs were plated onto fibronectin-coated coverslips and stained with phalloidin for F-actin or with antibody for vinculin. A typical micrograph for each condition including a magnification of an area of interest is shown. Bars equal 50 μm.

When primary SMC cultures are established, they initially vary in their extent of SMA expression but individual cultures then maintain the same level of SMA expression for up to 12 passages (unpublished observations). To overexpress SMA using a bicistronic lentiviral vector with GFP, a carotid SMC culture with relatively low endogenous SMA expression was used. Following viral infection and subsequent selection of cells expressing GFP, two batches of SMCs either expressing GFP alone (GFP-controls) or additionally over-expressing SMA (SMA-SMCs) were established. SMA-SMCs express approximately 5 times more SMA protein than controls (Fig 1A). As ectopically expressed SMA is VSVG-tagged, the tag is only found in SMA-SMCs (Fig 1A). When plated onto fibronectin, both controls and SMA-SMCs formed stress fibers and focal adhesions to comparable extent (Fig 1B). Accordingly, quantification of fluorescence signals in these experiments show no significant differences between GFP-controls and SMA-SMCs (S1 Fig).

**Fig 2:**
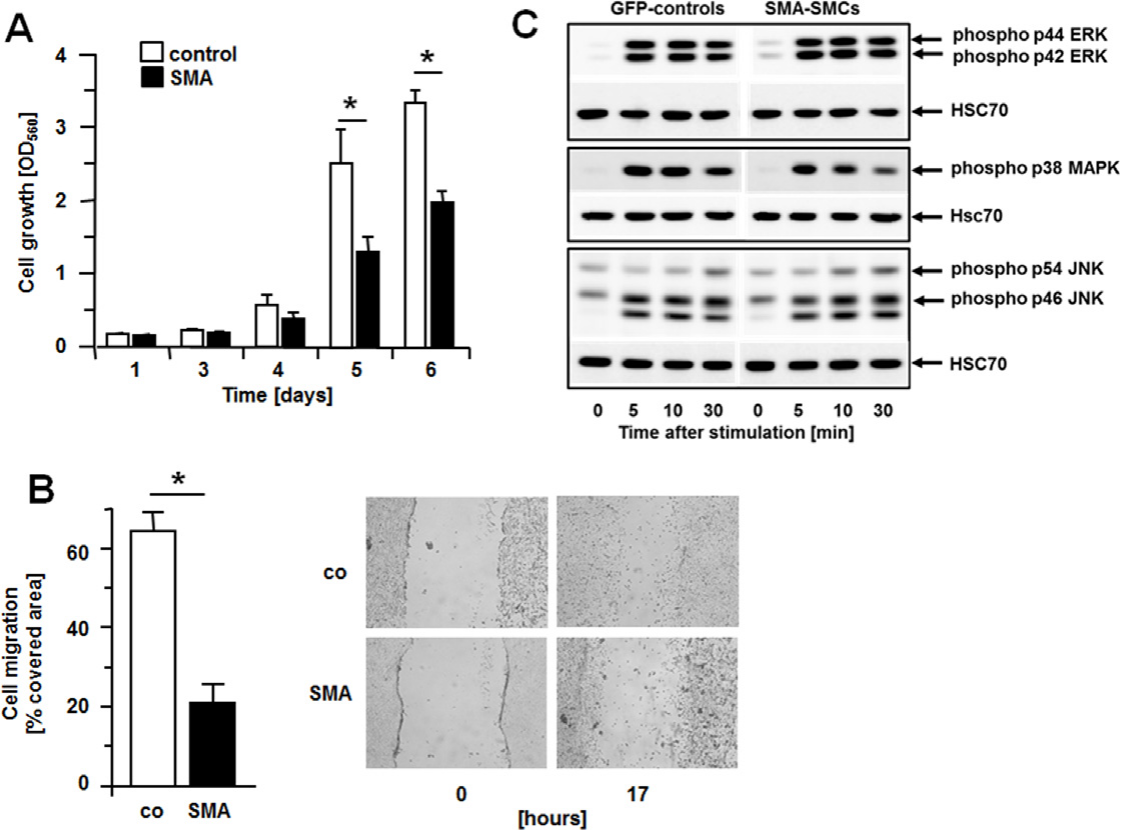
Expression of SMA inhibits SMC proliferation and migration but does not affect activation of MAPKs. (A) Proliferation of GFP-controls or SMCs overexpressing SMA was determined by MTT assays at various time points after stimulation with 10% FBS. Data (OD_560_ measurements) are presented as mean +/- S.D. of 6 independent experiments *P< 0.001. (B) Cell migration in response to 10% FBS was measured by scratch assays. Hydroxyurea at 5 mmol/L was added to prevent proliferation. Data (percent area covered by cells 17 hours after wounding) are presented as mean +/- S.D. of 6 independent experiments. *P<0.001. Representative micrographs for GFP-controls (co) and SMA-SMCs (SMA) are shown at time 0 right after wounding and at the end of the experiment. (C) Serum-starved cells (GFP-controls and SMA-SMCs) were stimulated with 10% FBS and harvested at the time points indicated. Cell extracts were subjected to Western blot analysis and blots probed with antibodies to recognize the phosphorylated forms of p44/p42 ERK, p38 MAPK or p54/p46 JNK. In all cases, HSC70 was used as a loading control. For each phospho-antibody, a representative result from three independent experiments is shown. The band below phospho-p46 JNK is considered a singularly phosphorylated form that does not shift, or a breakdown product.

Proliferation of GFP-controls and SMA-SMCs was measured using a metabolic labeling assay [17]. Compared to GFP-controls, SMA-SMCs proliferate considerably slower; at day 5 after initially plating the same number of cells, control cells increased 12-fold but SMA-cells only 6-fold (Fig 2A). An even stronger inhibition of cell function by SMA was observed in wound healing assays, where cells are removed form a confluent cell layer by scratching the well with a pipette tip and the area repopulated by cells is then measured after 17 hours. While control cells repopulated about 60% of the scratched area, SMA-SMCs only reached 20% (Fig 2B). As these assays were performed in the presence of the DNA synthesis inhibitor hydroxyurea, wound closure is considered a measure for cell migration. Thus, overexpression of SMA inhibits both SMC migration and proliferation. To investigate a possible involvement of MAPK activation in the SMA-mediated inhibition of SMC migration and proliferation, we measured phosphorylation of p44/p42 ERK, p38 MAPK and p54/p46 JNK in GFP-controls as well as in SMA-SMCs. No differences between the two cell types were found following stimulation with 10% FBS for 0–30 min (Fig 1C).

**Fig 3:**
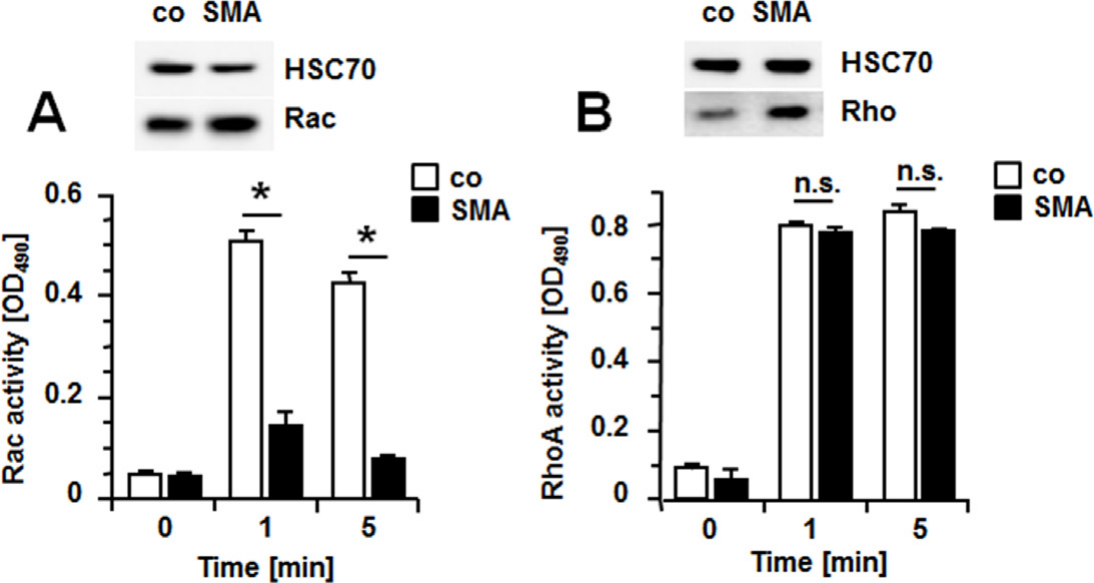
SMA inhibits serum-induced activation of Rac. Quiescent cells (GFP-controls or SMCs overexpressing SMA) were stimulated with 10% FBS for 1 and 5 min. Cell lysates were prepared and the amount of GTP-bound Rac (A) and Rho (B) determined by ELISA. All assays were performed in triplicates with two independently prepared cell batches for each condition. Data (OD_490_ measurements) are presented as mean +/- S.D. *P<0.001, n.s. = non-significant. Inserts show Western blots for total Rac and Rho protein in extracts of GFP-controls and SMA-SMCs. HSC-70 was used to confirm equal protein loading.

As small Rac and Rho regulate cell migration and proliferation, activation of these small GTPases was compared between controls and SMA overexpressing SMCs. Activities of Rac and RhoA were determined by measuring their GTP-bound form. One minute after serum stimulation of control SMCs, activities of Rac and RhoA were increased 10 and 8-fold, respectively (Fig 3). In SMA-SMCs however, Rac was only stimulated 3-fold, while stimulation of RhoA was to the same extent as in controls (Fig 3). The same pattern was observed at 5 min after stimulation (Fig 3). These results clearly show that increased expression of SMA reduces activation of Rac but does not affect RhoA activity. Notably, Western blot analysis revealed that when normalized to total cellular protein, GFP-controls express less Rac and Rho compared to SMA-SMCs (Fig 3 inserts).

**Fig 4:**
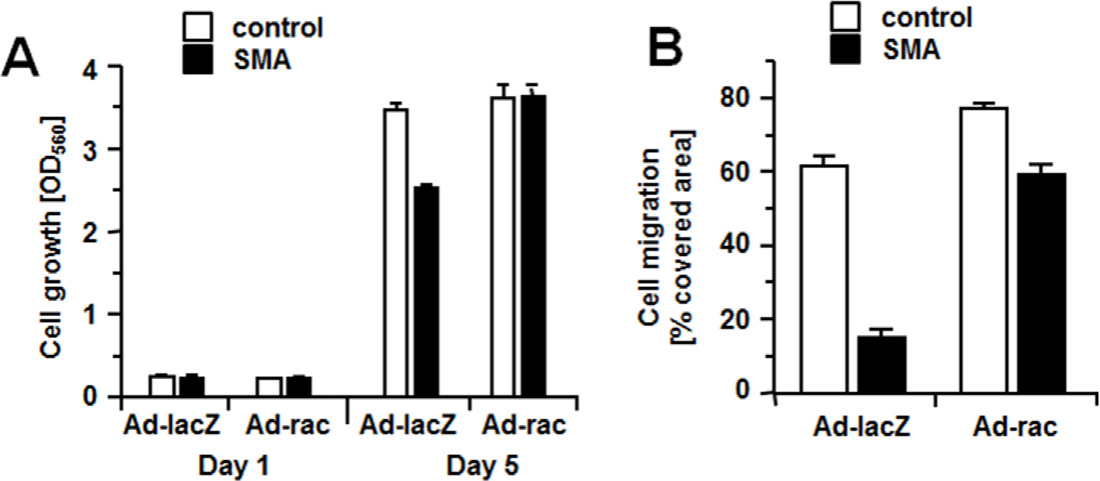
Expression of a constitutively active Rac1 mutant counteracts the inhibitory effects of SMA on cell migration and growth. Controls or SMCs overexpressing SMA were transfected with adenovirus to express lacZ (Ad-lacZ) or constitutively active V12rac1 (Ad-rac). All assays were performed in triplicates. (A) Proliferation of cells was determined by MTT assays at day 1 and day 5 after stimulation with 10% FBS. Data (OD_560_ measurements) are presented as mean +/- S.D. (B) Cell migration in response to 10% FBS was measured by scratch assays. Hydroxyurea at 5 mmol/L was added to prevent proliferation. Data (percent area covered by cells 17 hours after wounding) are presented as mean +/- S.D.

To link the inhibitory effects of SMA overexpression on SMC proliferation and migration to the decreased activation of Rac1, controls and SMA-SMCs were transfected with adenoviral vectors expressing lacZ or a constitutively active Rac1 mutant (V12rac1) and their proliferation and migration in response to serum was determined. In the presence of lacZ, SMA-SMCs proliferate less than controls, although the difference between the two cell types is smaller than has been observed prior to adenoviral transfection (compare Figs 2A and 4A). However, when V12rac1 is present, no difference between both cell types regarding proliferation is observed and both cell types proliferate as fast as controls expressing lacZ (Fig 4A). Expression of lacZ has no effect on the extent of inhibition of cell migration by ectopic SMA expression (compare Figs 2B and 4B). In contrast, when V12rac1 is present, SMC migration is significantly less inhibited by SMA overexpression (Fig 4B). The observations that overexpression of constitutive Rac1 rescues reduced proliferation and migration of SMA-overexpressing SMCs strongly indicate that SMA reduces proliferation and migration by inhibiting Rac1 activation.

**Fig 5:**
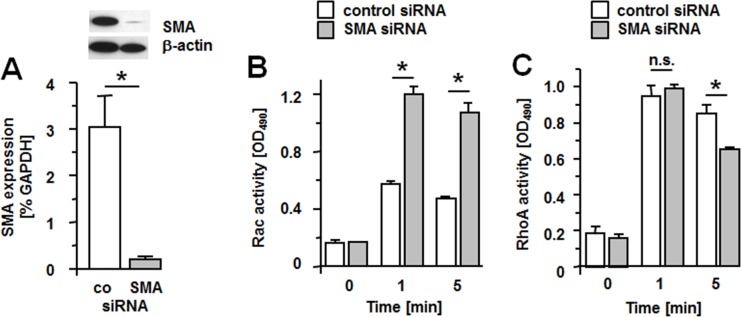
Knock-down of SMA expression increases the activation of Rac. SMCs were transfected with non-specific, GC-matched siRNA (control) or siRNA targeting SMA (SMA). (A) Expression of SMA was determined by qPCR measurements and normalized to GAPDH expression. Data (mean + S.D.) from 8 independent transfection experiments are shown. Insert: Western blot analysis of SMA protein expression. The blot was reprobed with beta-actin antibody as a loading control. (B, C) Quiescent controls or cells transfected with SMA-siRNA were stimulated with 10% FBS for 1 and 5 min. Cell lysates were prepared and the amount of GTP-bound Rac (B) and RhoA (C) determined by ELISA following the manufacturer’s instructions. Assays were performed in triplicates with two independent cell batches for each condition. Data (OD490 measurements) are presented as mean +/- S.D. *P<0.001, n.s. = non-significant.

Finally, we examined if down-regulation of SMA expression opposite the effect of SMA overexpression on Rac1 activation. For this purpose we selected a SMCs preparation with high basal SMA expression to treat with siRNA directed against SMA. This typically resulted in a reduction of SMA mRNA and protein by 90–95% (Fig 5A). SMCs with reduced SMA expression show an increased activation of Rac when compared to controls with more than twice as active Rac measured at 1 and 5 min after stimulation of cells with 10% FBS (Fig 5B). Both cell types activate RhoA to the same extent at 1 min after stimulation; at 5 min, SMCs with reduced SMA expression, however, had significantly lower GTP-RhoA levels than controls (Fig 5C). These results confirm that SMA negatively regulates activation of Rac1.

## Discussion

The SMC phenotype in healthy arteries is defined by the expression of SMA and other structural proteins including smooth muscle myosin heavy chain, SM22alpha and calponin-1. Expression of these smooth muscle genes is regulated by serum-response factor in combination with a member of the myocardin family of transcriptional coactivators such as myocardin or myocardin-related transcription factors (MRTFs) [19,20]. In diseased arteries or upon arterial injury, SMCs change their phenotype in that they decrease expression of smooth muscle genes and become responsive to chemokines and growth factors; this phenotypical switch depends on replacement of the SRF cofactor of the myocardin family with an Ets-domain containing ternary growth factor [3–6]. This process is in part regulated by actin dynamics as G-actin binds and sequesters MRTFs, which are released upon actin polymerization [21]. By this mechanism, RhoA-dependent stress fiber formation promotes expression of smooth muscle genes. As in vivo, loss of expression of smooth muscle genes in arteries is paralleled by increased SMC migration and proliferation, it is obvious to assume that SMC genes such as SMA directly inhibit SMC migration and proliferation. Surprisingly little, however, is known about this possibility. Early evidence that SMA inhibits cell migration and proliferation stems from work with tumor cells [9,10] and fibroblasts [11]. Only recently, SMA-null mice have been subjected to carotid ligation to study the arterial injury response [8]. These animals exhibit increased neointimal growth when compared to wild-type mice. SMCs derived from SMA-null aortas show increased expression of other smooth muscle genes as well as increased expression of PDGFRbeta. Moreover, compared to wild-type cells, these cells also show increased phosphorylation of focal adhesion kinase, increased basal activity of Rac1, increased phosphorylation of ERK, AKT and p70S6 kinases well as decreased levels of the cell cycle inhibitor p27, all of which may contribute to excessive neointimal hyperplasia observed in ligated SMA-null carotid arteries. The finding that SMA-null SMCs derived from SMA-null mice show increased expression of other smooth muscle genes may indicate that compensatory mechanisms in development exist to counteract the lack of SMA. To further address the question, which signaling elements are regulated by SMA, we chose to overexpress SMA in wild-type SMCs. At first sight, no obvious differences between controls and SMA-SMCs regarding formation of actin fibers or focal adhesions were observed (Fig 1) but SMA-SMCs migrate and proliferate less than controls (Fig 2A and 2B) showing an inhibitory effect of SMA on these processes. Comparing controls with SMA-SMCs regarding various signaling pathways including MAPKs (Fig 2C) identified Rac as a target for regulation by SMA. Serum-induced activation of Rac is significantly blunted in SMA-SMCs (Fig 3A). In contrast, activation of RhoA, which was measured in the same extracts, is not affected by SMA over-expression (Fig 3B). Although both GFP-controls and SMA-SMCs originated from the same parental SMC isolation, after multiple passages, GFP-controls express less Rac and Rho than SMA-SMCs. Up to date, it is unclear whether this is due to expression of the transgenes. Nevertheless, despite expressing more Rac protein than GFP controls, SMA-SMCs exhibit a decreased activation of Rac. Rac and Rho belong to the Rho family of small GTPases, which are involved in the regulation of virtually all cellular processes [22–26]. These proteins acquire their active conformation when GTP is bound and they can interact with and regulate downstream signaling molecules. Conversely, when GDP is bound, they in an inactive conformation, unable to interact with effector molecules. The major regulators of small GTPases are activatory proteins that promote the exchange from GDP to GTP (GEFs) and inhibitory proteins that promote GTP hydrolysis (GAPs).

The requirement for Rac1 in cell migration lies in the formation of cell protrusions at the leading edge of migrating cells [22,26]. There are multiple possibilities of how Rac1 may promote cell proliferation. Rac1 may increase expression of cyclinD1 thereby promoting G1/S progression in the cell cycle [24,25]. An early observation that cells expressing dominant negative Rac1 arrest in G2/M [27] indicated a role for Rac1 in mitosis. Interestingly, activated Rac1 accumulates in the nucleus at late G2 and forced expression of activated Rac1 in the nucleus increases the mitotic rate [28]. It should be noted that we measured all isoforms of Rac (Rac1, Rac2 and Rac3) and therefore, do not know the contribution of either isoform to total Rac activity. Although our observation that overexpression of constitutively active Rac1 (V12rac1) rescues cells from SMA-mediated inhibition of proliferation and migration (Fig 4) confirms a role for Rac1, it is a future task to investigate whether other Rac isoforms are involved as well. To demonstrate that also endogenous SMA inhibits serum-induced activation of Rac, we utilized siRNA technology to suppress expression of endogenous SMA (Fig 5A). Compared to controls, activation of Rac is significantly enhanced in SMA-siRNA-treated SMCs compared to controls (Fig 5B). Conversely, the activation of RhoA at 5 min is significantly lower in SMCs with suppressed SMA expression. (Fig 5C). It is possible that in these experiments, hyperactivation of Rac causes an inhibition of RhoA as such mechanism has previously been described [29].

Taken together, these findings suggest that activation of Rac is regulated by SMA expression. The observation that SMCs from SMA-null mice exhibit slightly increased basal Rac1 activity [8] supports the possibility that SMA-mediated suppression of Rac1 activation contributes to keeping medial SMCs quiescent in healthy arteries. Future work is now needed to study the molecular mechanisms of SMA-mediated inhibition of Rac. Likely candidates are Rac-GEFs that do not activate RhoA such as TIAM or RasGRFL [30,31].

## Conclusions

The overall conclusion of this work is that SMA expression negatively regulates the activation of Rac. This mechanism may contribute to keeping vascular SMCs quiescent in healthy arteries, where SMA expression is high. When SMA expression declines after arterial injury, hyper-activation of Rac may occur and promoting SMC migration and proliferation, thereby stimulating intimal hyperplasia.

## Supporting Information

S1 Fig Quantification of stress fibers and focal adhesions in controls and SMA-SMCs. GFP-controls (co) or SMA-SMCs (SMA) were plated onto fibronectin-coated coverslips and incubated with Alexa Fluor 568-coupled phalloidin to visualize F-actin (A) or with antibody for vinculin followed by an Alexa Fluor 568-conjugated anti-rabbit antibody to visualize focal adhesions (B). Intensity of fluorescence signals per area was calculated using ImageJ software (NIH). Exposure time was equal between different samples. Data (mean+/-S.D.) are shown for of three independent experiments with an evaluation of 29–63 individual cells per experiment and condition. Statistical significance was tested by t-test. n.s. = non-significant.
(TIF)
