# Supplementary figures and images for: Smooth Muscle-Alpha Actin Inhibits Vascular Smooth Muscle Cell Proliferation and Migration by Inhibiting Rac1 Activity
Source: PLoS One. 2016 May 13;11(5):e0155726. doi: 10.1371/journal.pone.0155726 (PMC4866761; doi:10.1371/journal.pone.0155726)

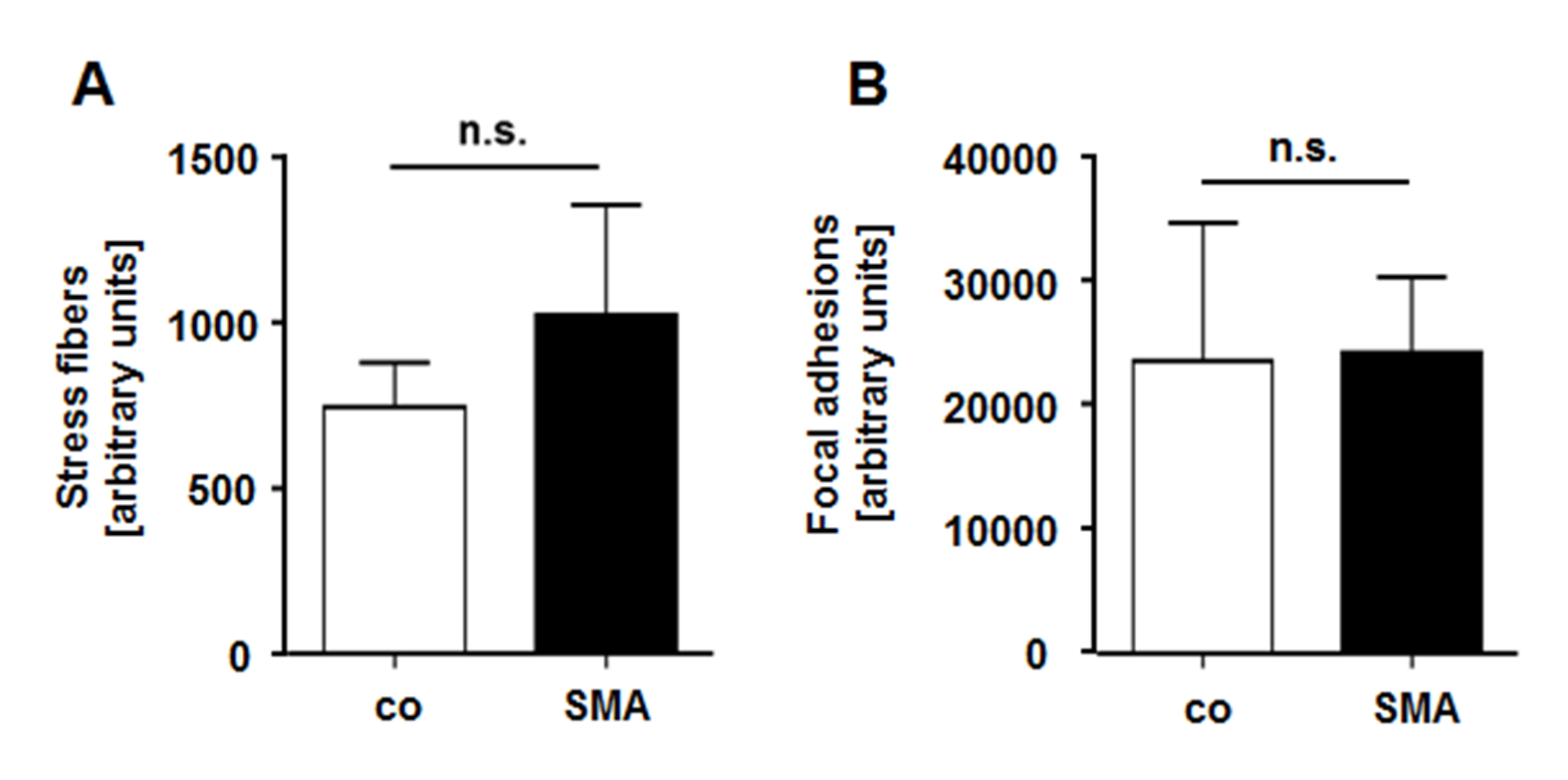

Supplement: S1 Fig — GFP-controls (co) or SMA-SMCs (SMA) were plated onto fibronectin-coated coverslips and incubated with Alexa Fluor 568-coupled phalloidin to visualize F-actin (A) or with antibody for vinculin followed by an Alexa Fluor 568-conjugated anti-rabbit antibody to visualize focal adhesions (B). Intensity of fluorescence signals per area was calculated using ImageJ software (NIH). Exposure time was equal between different samples. Data (mean+/-S.D.) are shown for of three independent experiments with an evaluation of 29–63 individual cells per experiment and condition. Statistical significance was tested by t-test. n.s. = non-significant. (TIF) [file pone.0155726.s001.tif]
